# External validation of the Toronto hepatocellular carcinoma risk index in a Swedish population

**DOI:** 10.1016/j.jhepr.2021.100343

**Published:** 2021-08-08

**Authors:** Hanne Åström, Nelson Ndegwa, Hannes Hagström

**Affiliations:** 1 Department of Medicine, Huddinge, Karolinska Institutet, Stockholm, Sweden; 2 Department of Medical Epidemiology and Biostatistics, Karolinska Institutet, Stockholm, Sweden; 3 Division of Surgery, Department of Clinical Science Intervention and Technology, Karolinska Institutet, and Oesophageal and Gastric Cancer Unit, Karolinska University Hospital, Stockholm, Sweden; 4 Division of Hepatology, Department of Upper GI, Karolinska University Hospital, Stockholm, Sweden; 5 Clinical Epidemiology Unit, Department of Medicine, Solna, Karolinska Institutet, Stockholm, Sweden

**Keywords:** Hepatocellular carcinoma (HCC), Toronto hepatocellular carcinoma risk index (THRI), Validation, ALD, alcohol-related liver disease, HCC, hepatocellular carcinoma, HR, hazard ratio, MELD, model for end-stage liver disease, NAFLD, non-alcoholic fatty liver disease, SVR, sustained virological response, THRI, Toronto HCC risk index, T2DM, type 2 diabetes mellitus

## Abstract

**Background & Aims:**

The Toronto hepatocellular carcinoma (HCC) risk index (THRI) is a predictive model to determine the risk of HCC in patients with cirrhosis. This study aimed to externally validate the THRI in a Swedish setting to investigate whether it could identify patients not requiring HCC surveillance.

**Methods:**

From 2004-2017, 2,491 patients with cirrhosis at the Karolinska University Hospital were evaluated. Patients were classified into low-, intermediate- and high-risk groups for future HCC according to the THRI. Harrell’s C-index, calibration-in-the-large, calibration slope and goodness-of-fit estimates were calculated to assess model discrimination and calibration. Cox proportional hazards regression was used to determine the risk of HCC.

**Results:**

Most patients were male (n = 1,638, 66%). The most common etiologies of cirrhosis were steatohepatitis (n = 1,182, 48%) followed by viral hepatitis (n = 987, 40%). In all, 131 patients (5.3%) were designated as low risk for HCC. Harrell’s C-index was 0.69. Calibration-in-the-large (0.11), calibration slope (1.24, not different from 1, *p* = 0.66) and goodness-of-fit showed good model calibration. Patients in the high-risk group had a 7.1-fold (95% CI 2.9–17.2) higher risk of HCC and patients in the intermediate-risk group had a 2.5-fold (95% CI 1.0–6.3) higher risk compared to the low-risk group.

**Conclusions:**

In a Swedish setting, the THRI could differentiate between low- and high-risk of HCC development. However, because the low-risk group was relatively small (5.3%), the clinical applicability of the THRI could be limited.

**Lay summary:**

The Toronto hepatocellular carcinoma (HCC) risk index (THRI) is a novel prediction model used to stratify patients with cirrhosis based on future risk of HCC. In this study, the THRI was validated in an external cohort using the TRIPOD guidance. Few patients were identified as low-risk, and the THRI had a modest discriminative ability, limiting its clinical applicability.

## Highlights

• The THRI is a simple and non-invasive method to estimate 5- and 10-year HCC risk.
• This was the largest validation of the THRI to date.
• The THRI had a modest discriminative ability and was well-calibrated.
• However, the THRI could only identify few patients at low risk of HCC, limiting its clinical use.

## Graphical abstract

**Figure:**
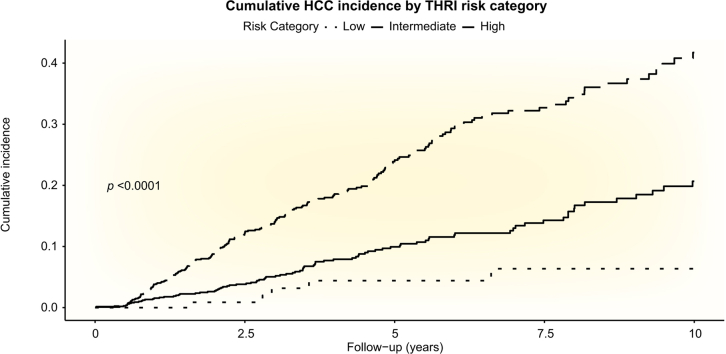

## Introduction

Cirrhosis, the most advanced form of liver disease, is the primary risk factor for hepatocellular carcinoma (HCC) development.^1^ Most cases of HCC are identified in patients with pre-existing cirrhosis.^2^ Early HCC identification has been prognostically favourable, conferring lower mortality rates and better prognosis.^3^^,^^4^

International guidelines recommend that all patients with cirrhosis with Child-Pugh class A-B and patients on the liver transplant waiting list be offered biannual HCC screening with ultrasound examination.^4^^,^^5^ These recommendations are supported by observational cohort^3^^,^^4^ and cost-effectiveness[5], [6], [7] studies.

The incidence of HCC varies greatly among different groups of patients. In addition, multiple risk factors, such as the etiology of liver disease, age, sex, type 2 diabetes (T2DM) and smoking, have been widely described.[8], [9], [10], [11], [12] However, the current HCC screening practice does not acknowledge the individual risk of HCC development in patients with cirrhosis,^4^^,^^5^ possibly exposing low-risk patients to unnecessary screening and risk of overdiagnosis.^13^

Several studies have attempted to use established risk factors to create a risk stratification system that could identify patients at varying levels of risk for HCC development.^14^^,^^15^ Such risk-scoring systems could help decide when to initiate HCC surveillance, thereby saving scarce healthcare resources.^13^

The Toronto HCC risk index (THRI) is an example of an HCC risk score suggested to identify low-risk patients not requiring HCC screening.^14^ The THRI is based on age, etiology of cirrhosis, sex and platelet count.^14^ These parameters are assigned points based on the hazard ratios (HRs) for the individual parameters as calculated in the original publication^14^ and stratify patients as having low, intermediate or high risk of HCC development.

External validation of any prediction model is vital before incorporation into clinical practice. Herein, we used the THRI in a Swedish cohort to investigate whether it could identify patients with a low incidence of future HCC that do not benefit from HCC surveillance.

## Patients and methods

### Patients

This was a cohort study based on historical data from patients with cirrhosis at the Karolinska University Hospital, a tertiary-level hospital in Stockholm, Sweden, between 2004 and 2017. The study methodology was made similar to the original THRI study^14^ to enhance comparability.

### Diagnosis of cirrhosis and HCC

**Fig. 1:**
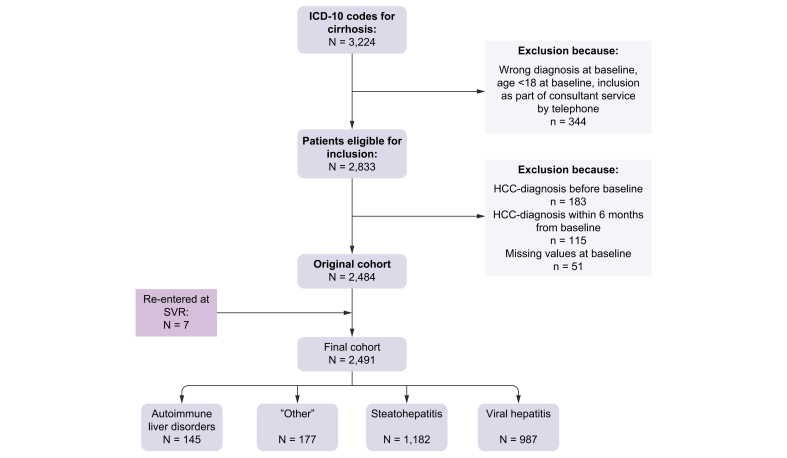
Flowchart of patient inclusion. HCC, hepatocellular carcinoma; SVR, sustained virologic response.

Patients were identified through the local electronic healthcare register, defining cirrhosis as an ICD-10 code (B180G, B181G, B182G, K70.3 or K74.6). The diagnosis of cirrhosis was then confirmed through a medical chart review. It was considered valid if any of the following criteria were met: signs of cirrhosis on pathology (*i.e.* biopsy with the presence of cirrhosis), radiology (surface nodularity, portal hypertension or fibroscan >14.5 kPa) or a clinical diagnosis (varices or ascites without any other explanation than cirrhosis) confirmed by a specialist in infectious diseases, hepatology or internal medicine. We excluded patients with a previous diagnosis of HCC or a diagnosis of HCC within 6 months from baseline. We also excluded patients for whom there was insufficient data to calculate the THRI. A flowchart for inclusion and exclusion is provided in Fig. 1. Patients meeting the criteria were included. Baseline was considered the date of the first recorded visit where the diagnosis of cirrhosis was established.

Patients were then divided into 4 groups based on the primary etiology of cirrhosis as in the original THRI paper^14^: (1) HCV *with* a previous sustained virological response (SVR) at baseline, or HCV *without* a previous SVR at baseline, or HBV.(2) Steatohepatitis (either non-alcoholic fatty liver disease [NAFLD] defined as a lack of clinically significant alcohol usage and either a BMI >30 kg/m^2^ or a BMI >25 kg/m^2^ and a co-existing diagnosis of T2DM; or alcohol-related liver disease [ALD] based on significant alcohol use identified by chart review or laboratory confirmation [phosphatidyl ethanol >0.3 μmol/L] and confirmed by a specialist in infectious diseases, gastroenterology or internal medicine).(3) Autoimmune liver disorders (autoimmune hepatitis, primary sclerosing cholangitis or primary biliary cholangitis).(4) “Other” (Wilson's disease, hemochromatosis, porphyria, alpha-antitrypsin deficiency, other rare genetic disorders and cryptogenic cirrhosis). If a patient had more than 1 etiology of cirrhosis, the etiological group was set according to the highest known risk of HCC development according to the THRI system.^14^ For instance, patients with both steatohepatitis (ALD or NAFLD) and viral hepatitis were assigned to the viral hepatitis group. The aim of this was to minimise inter-etiological confounding and establish consistency with the original THRI publication.^14^

During follow-up, the development of HCC was first established by an ICD-10 code of C22.0 present in the medical charts and verified on chart review. The method used to diagnose HCC was consistent with current guidelines^4^^,^^5^ and was formally made at a multidisciplinary tumour conference. The same electronic healthcare system is used in the Stockholm region, with the exception of 1 hospital (Saint Göran’s hospital). The Karolinska University Hospital is responsible for treating patients with HCC in the Stockholm area. Thus, we probably captured most incident HCCs in the cohort during follow-up.

### Outcomes and follow-up period

The end of follow-up was defined as either the date of HCC diagnosis, death, liver transplantation, loss to follow-up due to migration from the Stockholm region or after 10 years of follow-up, whichever occurred first. Patients who underwent treatment for HCV were censored at the time of SVR and then re-entered as a “new” patient based on the updated THRI score with new values for age, etiology and platelet count, if available at that date. Only complete-case analysis was considered and patients with missing data for any of the THRI values were censored at the date of SVR.

Age, sex, etiology of cirrhosis and platelet count were recorded at baseline to compute a THRI score. International normalized ratio, creatinine, bilirubin, sodium and dialysis-dependent kidney failure were additionally recorded to calculate a baseline model for end-stage liver disease (MELD) score. BMI measurements were deemed valid ±4 weeks from baseline.

### Statistical analysis

Analyses and external validation steps were performed on patients with complete data on the prognostic factors and outcome variables as defined by the development model. Predictors and outcome variables were defined in the same way or as close as possible to the development dataset.^14^

Moreover, the THRI risk score in the validation dataset was calculated using the values assigned to each predictor variable as defined in the development model. Briefly, for each patient in the cohort, a risk score was computed using the values in the development dataset. Based on these risk scores, patients were classified as having a low risk (<120), an intermediate risk (120-240) or a high risk (>240) of HCC.

HRs were estimated across HCC risk and etiologic groups using a Cox regression proportional hazards model. The low-risk and the autoimmune groups were set as a reference in the respective analyses. Both univariable and multivariable analyses, adjusted for the MELD score and T2DM at baseline, were performed.

The proportional hazards assumption was checked using scaled Schoenfeld residual plots and corresponding test statistics.^16^

A detailed description of the statistical method to externally validate the THRI is presented in the supplementary information. *p* <0.05 (2-sided) was considered statistically significant. Statistical analyses were performed in R Statistical software version 3.6.3 (R Foundation for Statistical Computing, Vienna, Austria) using the packages survival, rms and mice.

### Measures of discrimination and calibration

Two independent discrimination measures were estimated to evaluate model discrimination: the Harrell C-index^17^ and Royston and Sauerbrei’s R^2^_D_.^18^

Calibration was assessed by comparing the observed and predicted number of HCC events following Crowson’s method,^19^ which can be applied to the Cox proportional hazards model. Calibration-in-the-large, calibration slope and goodness-of-fit estimates were also calculated.

### Kaplan-Meier curves between risk groups

The incidence of HCC was determined for the THRI risk groups and etiological categories using the Kaplan-Meier method, and differences between groups were compared with the log-rank test.

Visual comparisons between these curves and those in the development dataset were also performed to provide a qualitative assessment of the calibration. When curves from the development and validation datasets were superimposable, qualitative calibration assessment was deemed successful.

### Annual and cumulative HCC incidences

To validate the discriminative capacity of the THRI 5- and 10-year cumulative and annual incidences of HCC were calculated for the THRI and etiological groups.

### Ethical considerations

This study was approved by the Regional Ethics Committee in Stockholm (dnr 2016/177231/2 and 2018/450-32). The Committee determined that informed consent was not required for this cohort study.

## Results

### Participants

In all, 3,224 patients were identified using ICD-10 codes for cirrhosis. After exclusion, the final sample included 2,491 patients (Fig. 1). Totally, 371 patients achieved SVR during follow-up. However, because of missing data on ≥1 of the THRI parameters (most commonly platelets that were not sampled at the department of infectious diseases per protocol at SVR), we were only able to recalculate the updated THRI value for 7 of these patients. Of these 7 patients, 2 developed HCC during the follow-up period. In this study 131 patients (5.3%) were defined as low, 1,109 (44.5%) as intermediate and 1,251 (50.2%) as high risk.

### Cohort characteristics

Baseline characteristics of the cohort are presented in Table 1. The most common cause of cirrhosis was steatohepatitis (n = 1,182, 47.5%), followed by viral hepatitis (n = 987, 39.6%) and “other” causes of cirrhosis (n = 177, 7.1%). Three hundred and four (12.2%) patients developed HCC during the first 10 years after baseline. Patients with viral hepatitis accounted for 55.6% of all HCC cases (n = 169) and those with steatohepatitis accounted for 37.5% of HCC cases (n = 114). The least common cause of HCC was autoimmune liver disorders (2%, n = 6).

**Table 1:** **Baseline characteristics of the entire cohort stratified on development of HCC during follow-up or not**.

Variable	N	No HCC (n, 2,187)	HCC (n, 304)	*p* value
Mean age, years (SD) (Range)	2,491	58.6 (11.3) (19-91)	60.9 (8.7) (33-83)	<0.001∗
Sex, n (%)	2,491			0.006∗∗
Female	853	770 (35.2)	83 (27.3)	
Male	1,638	1,417 (64.8)	221 (72.7)	
Mean follow-up, years (SD) (Range)		3.7 (3.1) (0-10)	3.3 (2.3) (0-10)	0.48∗
Etiology, n (%)	2,491			<0.001∗∗
Viral†	987	818 (39.6)	169 (55.6)	
HBV	102	86 (3.9)	16 (5.3)	
HCV-SVR	109	104 (4.8)	5 (1.6)	
HCV-no SVR	801	649 (29.7)	152 (50.0)	
Steatohepatitis	1,182	1,068 (48.8)	114 (37.5)	
ALD	950	870 (39.8)	80 (26.3)	
NAFLD	238	204 (9.3)	34 (11.2)	
Autoimmune†	145	139 (6.4)	6 (2.0)	
AIH	126	116 (5.3)	10 (3.3)	
PBC	33	32 (1.5)	1 (0.3)	
PSC	33	32 (1.5)	1 (0.3)	
Other	177	162 (7.4)	15 (4.9)	
Mean BMI, kg/m^2^ ± SD (range)		27.2 ± 5.8 (14-57)	27.4 ± 4.8 (18-48.5)	0.28∗
T2DM (%)	620	525 (24.0)	95 (31.3)	0.006∗∗
MELD ± SD		11.9 ± 5.1 (6.4-38.4)	10.3 ± 3.0 (6.4-23.6)	0.003∗
Platelets, 10^9^/L (95% CI)	2,491	153	125	<0.001∗∗

AIH, autoimmune hepatitis; ALD, alcohol-related liver disease; HCC hepatocellular carcinoma; MELD, model of end-stage liver disease; NAFLD, non-alcoholic fatty liver disease; PBC, primary biliary cholangitis; PSC, primary sclerosing cholangitis; SVR, sustained virologic response; T2DM, type 2 diabetes.

∗ Mann-Whitney *U* test

∗∗ Chi-squared test.

† Overlap between diseases in a proportion of patients.

Compared to patients who did not develop HCC, those that did were older (mean 60.9 *vs*. 58.6 years-old, *p* <0.001) and had a higher prevalence of T2DM (31.3% *vs.* 24.0%, *p* = 0.006). Patients who developed HCC were primarily male (HCC 72.7% *vs.* no HCC 64.8%, *p* = 0.006) and had a lower baseline MELD score (10.3 *vs.* 11.9, *p* = 0.003, respectively).

### External validation

#### Measures of discrimination

A Harrell’s C-index of 0.69 of our model was obtained and an identical value of 0.69 was yielded using Royston and Sauerbrei’s R2D measure. Both measures indicate modest discrimination of the model.

#### Measures of calibration

A calibration-in-the-large value of 0.11 was obtained (which is not different from 0), indicating no evidence of global miscalibration of the model. The model's calibration slope was 1.24, a value not different from 1 (*p* = 0.66). The goodness-of-fit test indicated no overall evidence of the prognostic index's lack of fit in the validation data. Collectively, these results indicate good model calibration in the external validation data.

#### Assessment of model misspecification

No violation of the proportional hazards assumption was found using scaled Schoenfeld residuals (chi-squared 4.92, *p* = 0.43) on the validation dataset.

#### Kaplan-Meier curves between risk groups

**Fig. 2:**
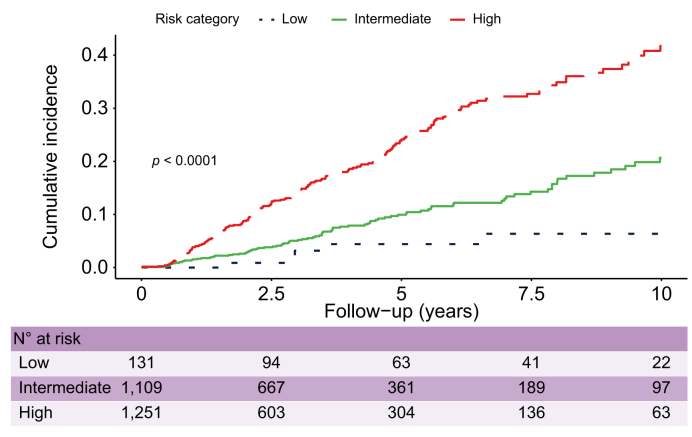
Kaplan-Meier curves for HCC development according to THRI groups. Cumulative incidence is illustrated and compared for the 3 THRI groups. The x-axis describes years of follow-up and the y-axis represents the proportion of patients with HCC. Curves were compared using the log-rank test (*p* <0.0001). HCC, hepatocellular carcinoma; THRI, Toronto HCC risk index.

An assessment of the Kaplan-Meier curves (Fig. 2) for the HCC risk groups provides informal visual evidence of discrimination as the curves were well separated, further supported by a formal comparison using the log-rank test (*p* <0.01). The risk of HCC was highest in the high-risk category and lowest in the low-risk category.

**Fig. 3:**
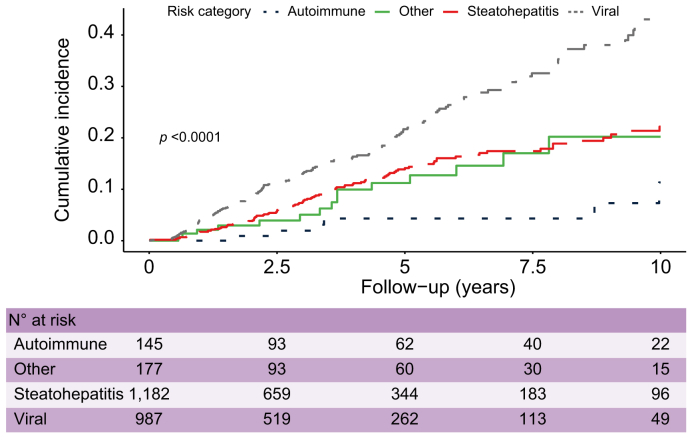
Kaplan-Meier curves for HCC development in the etiological groups. Cumulative incidence is illustrated and compared for the 4 etiological subgroups. The x-axis describes years of follow-up and the y-axis the proportion of patients with HCC. Curves were compared using the log-rank test (*p* <0.0001). HCC, hepatocellular carcinoma.

The Kaplan-Meier curves for the etiological groups (Fig. 3) illustrate the proportion of patients in each etiological group (autoimmune, steatohepatitis, viral, “other”) with HCC during follow-up. Patients with viral hepatitis had a higher rate of HCC during follow-up than the other etiological groups. The “other” and steatohepatitis curves intersected at multiple points and were not well differentiated from each other.

#### Hazard ratios between risk groups

HRs for the development of HCC according to the THRI and the etiological risk groups are presented in Table 2.

**Table 2:** **Cox regression analysis of THRI and etiological groups**.

Factors associated with HCC	Univariable HR (95%CI)	*p* value∗	Multivariable HR (95%CI)	*p* value∗
THRI group				
Low	1.0 (ref)	–	1.0 (ref)	–
Intermediate	2.8 (1.1–7.0)	0.024	2.5 (1.0–6.3)	0.042
High	7.3 (3.0–17.7)	<0.001	7.1 (2.9–17.2)	<0.001
Etiological group				
Autoimmune	1.0 (ref)	–	1.0 (ref)	–
Steatohepatitis	3.0 (1.3–6.9)	0.008	2.5 (1.1–5.8)	0.027
Viral hepatitis	5.8 (2.6–13.1)	<0.001	5.2 (2.3–11.8)	<0.001
“Other”	2.6 (1.0–6.8)	0.045	2.5 (1.0–6.5)	0.062

Cox regression analysis of the THRI and etiological groups. Adjusted for MELD score and T2DM in the multivariable analysis. HCC hepatocellular carcinoma; MELD, model of end-stage liver disease; T2DM, type 2 diabetes; THRI Toronto HCC risk index.

∗ Chi-squared test.

For the THRI groups, the rate of HCC increased in the intermediate- and high-risk groups, with the rate rising in a dose-response relationship. The risk group results remained statistically significant after adjusting for T2DM and MELD in the multivariable analysis (intermediate-risk group: adjusted HR [aHR] 2.5, 95% CI 1.0–6.3; high-risk group: aHR 7.1, 95% CI 2.9–17.2, Table 2).

Compared to patients with cirrhosis due to autoimmune liver disease, the risk of HCC development was highest in the viral group (aHR 5.2, 95% CI 2.3–11.8). A significantly higher risk was also seen in the steatohepatitis group (aHR 2.5, 95% CI 1.1–5.8), but not in the “other” group (aHR 2.5, 95% CI 1.0–6.5) (Table 2).

#### Annual and cumulative HCC incidence

Table 3 shows annual and cumulative incidences at 5 and 10 years, as well as overall incidence during the full follow-up period. The highest 10-year annual and cumulative incidence of HCC per 1,000 person-years in the 3 THRI risk groups was observed in the high-risk group (annual: 51, 95% CI 45–59; cumulative: 41.7, 95% CI 36.0–48.0), followed by the intermediate-risk group (annual: 20, 95% CI 17–25; cumulative: 20.7, 95% CI 16.3–25.9). The lowest 10-year HCC incidences were observed in the low-risk group (annual: 7, 95% CI 3–18; cumulative: 6.4, 95% CI 2.6–15.2).

**Table 3:** **Annual and cumulative incidences of HCC per 1,000 person-years in the THRI and etiological groups**.

	N at baseline (%)	Annual incidence at 0-5 years (95% CI)	Annual incidence at 0-10 years (95% CI)	Cumulative incidence at 0-5 years (95% CI)	Cumulative incidence at 0-10 years (95% CI)	Overall incidence per 1,000 person-years (95% CI)†
Full cohort	2,491	33 (29–38)	33 (30–37)	16.3% (14.3–18.4)	29.1% (23.7–32.9)	33 (30–37)
THRI score						
Low	131	8 (3–22)	7 (3–18)	4.4% (1.7–11.4)	6.4% (2.6–15.2)	7 (3–18)
Intermediate	1,109	19 (15–25)	20 (17–25)	10.0% (7.8–12.6)	20.7% (16.3–25.9)	20 (17–25)
High	1,251	51 (44–59)	51 (45–59)	24% (20.9–27.7)	41.7% (36.0–48.0)	51 (45–59)
Etiology						
Autoimmune	145	8 (3–22)	9 (4–20)	4.3% (1.6–11.1)	11.3% (4.5–27.0)	9 (4–20)
Steatohepatitis	1,182	28 (23–34)	26 (22–31)	14.1% (11.6–17.1)	22.2% (18.1–27.1)	26 (22–31)
Viral Hepatitis	987	46 (39–55)	49 (42–57)	21.7% (18.4–25.5)	43.1% (36.5–50.2)	49 (42–57)
“Other”	177	22 (12–40)	23 (14–38)	11.2% (6.3–19.7)	20.2% (11.9–33.3)	23 (14–38)
Virological status						
HCV-no SVR	801	47 (39–56)	50 (43–59)	24.8% (20.9–29.4)	50.9% (42.8–59.6)	59 (50–69)
HCV-SVR‡	109	13 (5–30)	10 (4–24)	6.0% (2.5–14.0)	6.0% (2.5–14.0)	10 (4–24)

HCC, hepatocellular carcinoma; SVR, sustained virological response; THRI, Toronto HCC risk index.

† Entire follow-up period

‡ At baseline

Patients in the viral group had the highest annual and cumulative HCC incidences at 5 and 10 years. The second-highest cumulative and annual HCC incidences were seen in patients with steatohepatitis. Patients in the “other” group had a similar HCC incidence to patients with steatohepatitis. The lowest HCC incidence was observed in patients with cirrhosis due to autoimmune liver diseases (Table 3).

## Discussion

This study tried to externally validate the THRI to determine whether it could be used to identify patients who benefit from HCC screening. We identified 2,491 patients with cirrhosis, making this the largest validation study of the THRI. Compared to the lower-risk group, patients in the high-risk group had a 7.1-fold higher risk of HCC. The intermediate-risk group had a 2.5-fold higher risk of HCC than the lower-risk group. The cumulative incidence of HCC in the high-risk group at 10 years of follow-up was 42%, suggesting that this could be a subgroup of patients for which efforts should be made to ensure optimal HCC surveillance.

Thus, we confirm that the risk of HCC varies across groups of patients with cirrhosis, calling for individualised decisions on screening for HCC. However, the low-risk group was comparably small (only 5.3%), limiting the adaption of the THRI in clinical practice, also supported by the modest discrimination of the model (C-index = 0.69).

According to the TRIPOD criteria, the THRI in the current cohort was assessed using model discrimination and calibration.^20^ Overall, the performance of the THRI in this Swedish cohort was mediocre, largely due to its limited discriminative ability, implicating that it could not adequately differentiate between the patients with and without HCC development during follow-up. However, the model was well-calibrated, *i.e.* the predicted risk conformed to the observed risk of HCC development in the current cohort and did not under- or overpredict HCC development.

The THRI performed better in both the external and internal validation cohorts in the original publication (C-index in internal validation: 0.75, external validation: 0.77) compared to the current cohort (C-index = 0.69).^14^ This difference is likely due to the overall higher HCC incidences reported in our study.^14^

The THRI was also validated by Zhang *et al.* in a cohort of 520 Chinese patients in 2019.^21^ This study, comparable to ours, reported a higher incidence of HCC than Sharma *et al.*^14^ Zhang *et al.* suggested that this finding could result from the higher prevalence of HBV,^21^ as patients with HBV are generally at the highest risk of HCC development.[21], [22], [23] Similar to our findings, Zhang *et al.* reported that only a small proportion (4.4%) of the patients were considered low-risk according to the THRI.

The mean age of our cohort was higher at baseline than in Sharma *et al.*’s cohort (53.9 years *vs*. 58.9 years), which could explain the higher HCC incidence seen in this study as age is a major risk factor for HCC development.^24^ The Karolinska University Hospital is a referral centre for patients with severe cirrhosis, including evaluations for liver transplantation. This fact could partially explain the divergence in HCC incidence, especially as the severity of cirrhosis is a known risk factor for HCC development.^25^

Previous research on HCC epidemiology suggests that patients with untreated viral hepatitis, and HBV in particular, are at the greatest risk of HCC development.^2^^,^^8^ Therefore, it was unexpected that Sharma *et al.* reported a higher prevalence of HBV (4% in our study *vs.* 19% in Sharma *et al.*) but a lower HCC incidence for the viral group (10-year cumulative incidence in our study was 34% *vs.* 22% in Sharma *et al.*).^14^

Introducing a risk index (such as the THRI) into the regular follow-up of patients with cirrhosis could further individualise patient management. Even if the number of patients in the low risk group was low, such individuals can be reassured of a low risk and be excluded from surveillance, although repeated evaluations of the THRI would need to be made. Further, patients in the high-risk group are clearly at an elevated risk of HCC, so efforts should be made to make sure surveillance is optimal in these patients. They could also form the basis for future studies on the frequency of surveillance, such as a randomized controlled trial of screening every 3 months compared to current practice.

While the present results are insufficient to suggest a change in HCC surveillance in the current population, we recognise that future studies on HCC risk scoring and studies on the cost-effectiveness of HCC screening are warranted to minimise unnecessary diagnostics with uncertain cost-effectiveness and potential overdiagnosis.

The medical community has recognised the benefit of an HCC risk index. Indeed, there have been several attempts at creating an HCC risk index for patients with cirrhosis or at otherwise increased risk of HCC development.^15^^,^^26^^,^^27^ Because the THRI is a relatively simple risk index that would be easy to implement in clinical practice, we chose to validate this index. Nevertheless, we recognise that future validation of other HCC risk indexes is warranted.

The current validation found that the modest performance of THRI could not identify a larger low-risk group. However, we did find that both the low-risk and the autoimmune group presented with an annual HCC incidence lower than the previously suggested 1.5% threshold. This threshold was drafted by a few studies on the cost-effectiveness of HCC screening conducted on all patients with cirrhosis in which different etiologies of cirrhosis or other known risk factors were not considered. While this threshold was not intended for studying specific subgroups of patients, it indicates that some of these groups might not benefit from HCC screening.

This is further supported by some recent studies on HCC epidemiology showing that the risk of HCC development is relatively low in some unselected populations. Recently, Jepsen *et al.* and Hagström *et al.* independently reported low incidences of HCC in patients with ALD-related cirrhosis, suggesting that regular HCC screening in these patients might not be cost-effective.^28^^,^^29^

The main limitation of this study is that Karolinska University Hospital accepts referrals and coordinates the treatment of patients with severe and hard-to-treat cirrhosis. The severity of cirrhosis is commonly accepted as a risk factor for HCC development. Thus, selection bias may have led to a higher HCC incidence than that reported by Sharma *et al.*^14^ and a lower proportion of low-risk patients. However, this is a real-life cohort from a large tertiary centre. Thus, we should be able to generalise our results to similar settings. Due to missing data, we could not recalculate the THRI at the time of SVR in most patients with HCV cirrhosis. Finally, there were relatively few patients with autoimmune liver diseases, and future studies could strive to investigate the THRI in larger populations.

The THRI is a novel risk stratification system that questions the effectiveness of HCC surveillance in all populations. In this study we tried to validate THRI in a Swedish setting and found that the system could differentiate between low- and high-risk patients. However, it could only identify a small group of low-risk patients (5.3%), suggesting that it only has modest clinical applicability.

## Financial support

HH was supported by grants from Region Stockholm (clinical postdoctoral appointment), 10.13039/501100007232Radiumhemmets Forskningsfonder and The 10.13039/100012538Swedish Cancer Foundation.

## Authors’ contributions

Study conception and design: HH. Acquisition of data: HÅ. Statistical analysis: HH, NN.Analysis and interpretation of data: All. Drafting of the manuscript: HÅ, HH, NN.Critical revision: All. Guarantor of the article: HH. All authors approved the final version of the article, including the authorship list.

## Data availability statement

Due to the confidentiality of data, the data which support the findings of this study are generally not available due to current regulations. However, requests for additional analyses might be considered upon reasonable request.

## Conflict of interest

The authors declare no conflicts of interest that pertain to this work.

Please refer to the accompanying ICMJE disclosure forms for further details.
