# Supplementary material for: External validation of the Toronto hepatocellular carcinoma risk index in a Swedish population
Source: JHEP Rep. 2021 Aug 8;3(5):100343. doi: 10.1016/j.jhepr.2021.100343 (PMC8476346; doi:10.1016/j.jhepr.2021.100343)
Supplement: Multimedia component 1 [file mmc1.pdf]

External validation of the Toronto hepatocellular carcinoma risk index in a Swedish population

Hanne Åström, Nelson Ndegwa, Hannes Hagström

Table of contents

Supplementary materials and methods..2

Supplementary materials and methods

Upon our request to the THRI model authors, we obtained the baseline survival function S0(t) and their corresponding follow-up times. The SO(t) allowed for a facilitated stricter model calibration. We selected the baseline survival value closest to the follow-up amount for each patient and incorporated it into the validation model.

To prevent the extrapolation of the baseline survival curve beyond the observed range of t, we truncated the follow-up period in the validation dataset to the maximum t of the development dataset (approximately 10 years).

Two independent discrimination measures were estimated to evaluate model discrimination: the Harrell C-index (17) and Royston and Sauerbrei's R^2_D (18).

Harrell's C-index is defined as the proportion of all usable patient pairs for which the predictions and outcomes are concordant. A measure of 0.5 corresponds to flipping a coin with 1 reflecting perfect discrimination.

Royston and Sauerbrei's R^2D measures the proportion of explained variation based on D, a measure of a model's ability to discriminate between good and poor patient outcomes.

Calibration, the model's ability to predict the number of observed cases of HCC during follow-up, was assessed by comparing the observed and predicted number of HCC events following Crowson's method (19), which can be applied to the Cox proportional hazards model. Calibration-in-the-large, calibration slope and goodness-of-fit estimates were calculated.

Calibration-in-the-large quantifies agreement between the observed and predicted risks by comparing the mean of all predicted risks with the mean of all observed risks. Hence, it shows the extent to which predictions are systematically too high or too low. A value of 0 is ideal.

The calibration slope was calculated by regressing the observed outcome on the predicted probabilities. If the observed and predicted values match, the slope should equal 1. A likelihood ratio test was used to determine if the slope of the regression line is 1.

The goodness-of-fit test performs a comparison of observed and expected frequencies within risk strata. Participants were grouped into quintiles of predicted HCC risk during follow-up, which was calculated in the first step of the model. Using Poisson regression for survival data, the observed incidence of HCC was estimated within each quintile, taking into account the censored nature of the data (19). The deviance goodness-of-fit test for Poison regression is then performed, with the null hypothesis that the model is correctly specified.
